# Effects of a clinical pathway on antibiotic use in patients with community-acquired pneumonia: a multi-site study in China

**DOI:** 10.1186/s12879-018-3369-1

**Published:** 2018-09-19

**Authors:** Liping Zhu, Jie Bai, Yongcong Chen, Di Xue

**Affiliations:** 1 NHC Key Laboratory of Health Technology Assessment (Fudan University), Department of Hospital Management, School of Public Health, Fudan University, Shanghai, People’s Republic of China; 2 School of Public Health, Lanzhou University, Lanzhou, People’s Republic of China

**Keywords:** Clinical pathway, Antibiotic, Implementation, Community-acquired pneumonia

## Abstract

**Background:**

Community-acquired pneumonia (CAP) is a common condition with high mortality, morbidity and healthcare costs. This study aimed to determine whether clinical pathway (CP) implementation in different hospitals in China increased antibiotic compliance with the national CP in inpatients with CAP.

**Methods:**

Chart reviews of CAP cases were conducted in 18 public hospitals from 3 different regions of China in 2015. Chi-square tests and the t-test were used to compare differences between hospitals that implemented CP (CP group) and those that did not (non-CP group). Multivariate logistic analysis was adopted to test whether CP implementation for CAP in hospitals affected their overall antibiotic use compliance rates with the national CP for CAP.

**Results:**

The overall compliance rate with the national CP for inpatients with CAP was 43.69%. The compliance rates for timely initial antibiotic use, recommended antibiotic use and use of the recommended combination of antibiotics and the overall compliance rate were substantially higher in the CP group than in the non-CP group. A multivariate logistic model for overall compliance in inpatients with CAP showed that the hospitals in the CP group had greater overall compliance than those in the non-CP group (odds ratio [OR] = 1.76; 95% confidence interval [CI] = 1.16–2.71) after controlling for hospital and inpatient characteristics.

**Conclusion:**

In China, the overall compliance rate with the national CP for inpatients with CAP was low, but inpatients with CAP in the hospitals in the CP group received antibiotics more concordantly with the national CP. Since adherence to evidence-based care has been shown to improve clinical outcomes, internal and external support from hospitals is required to facilitate CP implementation for inpatients with CAP. Additionally, governmental commitment, hospital input and population involvement are required to improve antibiotic utilization.

**Electronic supplementary material:**

The online version of this article (10.1186/s12879-018-3369-1) contains supplementary material, which is available to authorized users.

## Background

Community-acquired pneumonia (CAP) is a common condition with high mortality, morbidity and costs [1] and is also the most common infectious cause of death worldwide [2]. Empiric antibiotic therapy is the cornerstone of CAP treatment. According to the guideline for inpatients with CAP outside of the intensive care unit (ICU), the preferred antibiotics are β-lactams* plus macrolides in the United States, amoxicillin plus macrolides in Britain and aminopenicillin with or without macrolides in Europe. Respiratory fluoroquinolones are alternative antibiotics in the CAP guidelines in all of these countries or regions [3–5]. Similar to many other countries, the national CAP guideline for inpatients with CAP outside of the ICU in China recommends the use of a β-lactam/β-lactamase inhibitor and a 2nd-generation cephalosporin, cefotaxime or ceftriaxone with or without macrolides; the alternative recommendation is application of a respiratory quinolone [6, 7].

However, inappropriate antibiotic use for CAP treatment has been well documented [8]. The World Health Organization (WHO) has reported that antibiotic resistance causes 2.50 thousand deaths annually in the European Union and 204.94 thousand illnesses and 2.30 thousand deaths annually in the United States [9]. Antibiotic resistance also has a significant economic impact due to the need for more drugs and longer hospital stays [10]. A large-scale study reported that antibiotic use was several-times higher in China than in other developing countries [11]. In 2010, 49.63% of patients in tertiary hospitals in China were prescribed antibiotics, and 32% of inpatients received antibiotic combination therapy [12]. Because of inappropriate antibiotic use, China has experienced the most rapid growth of antibiotic resistance worldwide [13]. *Clostridium difficile* infection (CDI) is another incidental complication related to antibiotic use [14] and has become a major public health problem associated with significant morbidity, mortality and hospital costs [15, 16].

With the increase in antibiotic resistance, the decline in antibiotic development and the frequent occurrence of adverse events, antibiotics should be used wisely for greater effectiveness and lower risks [17]. One strategy to address the antibiotic crisis is adoption of clinical guidelines (CGs) and clinical pathways (CPs) [18, 19]. Although both CGs and CPs have been embraced as strategies to decrease clinical practice variation, CPs, which are operational structures usually based on pre-existing CGs, are more explicit concerning the participants, sequence, timing and provision of interventions for patients with specific medical conditions [20–22]. In China, national CPs are issued by the National Health Commission (NHC) in accordance with national CGs. A total of 1010 CPs had been issued in China by the end of 2016 [23]. Hospitals in China can modify national CPs slightly to make them more practical for local use, and some hospitals require physicians to provide medical care that adheres to CPs (including recommended antibiotic use).

In 1988, the Infectious Diseases Society of America (IDSA) initially published CPs to improve antibiotic use in inpatients and to minimize or eliminate the increased morbidity, mortality and health care costs attributed to antibiotics [24]. Recently, a positive correlation between the implementation of CPs and improvement in antibiotic use was found in some studies in China [25, 26]. However, many previous studies on the effects of CPs on antibiotic use in China are limited to one hospital or by a small sample size. Therefore, testing the effects of CP implementation on general antibiotic use is difficult.

This study aimed to determine whether CP implementation in different hospitals from three representative regions of China increased antibiotic compliance with the national CP for inpatients with CAP.

## Methods

### Survey sample

This retrospective study was conducted in Shanghai, Hubei Province and Gansu Province, which represented high, middle and low socioeconomic status levels and the eastern, central and western regions of China in 2015, respectively. In the Hubei and Gansu provinces, 3 areas (cities or autonomous prefectures) were selected to represent high, middle and low socioeconomic status levels in each province. In each surveyed area of the Hubei and Gansu provinces, 1 tertiary and 1 secondary public hospital were selected as the surveyed hospitals. Because the tertiary public general hospitals in Shanghai were not evenly distributed among districts, 3 tertiary public general hospitals were selected in Shanghai to represent tertiary hospitals owned by a university, the Shanghai government and the district government. In Shanghai, 3 secondary public general hospitals were also selected from 3 districts to represent hospitals in urban, suburban and rural areas.

### Data sources

Chart reviews were conducted to collect information on antibiotic use from inpatients with CAP who were admitted to any of the 18 surveyed hospitals in 2014. We identified all inpatients with CAP admitted to each hospital in 2014 based on the International Statistical Classification of Diseases and Related Health Problems, 10th Revision (ICD-10) codes. Inpatients admitted to the ICU during their hospitalization were excluded from this study. For most hospitals, hospital electronic information systems were used to identify cases and to collect information from the first pages of medical records (including information regarding patient characteristics and diagnoses). However, for hospitals in rural areas without electronic health information systems, cases were identified, and information on the first pages of medical records was collected manually.

To ensure that the sample was evenly distributed throughout the year, 2–3 cases were selected each month; thus, 30 cases were sampled from each hospital. For the hospitals with electronic medical information systems, we selected 2–3 medical records per month with a random sampling method using the randomizing formula in Excel. For the hospitals without electronic medical information systems, we used the convenient sampling method to select 2–3 medical records per month. If a hospital admitted fewer than 30 inpatients with CAP in 2014, then all medical records from 2014 and some records from late 2013 or even all of 2013 were extracted so that 30 records could be extracted for each condition in each hospital.

We developed an audit chart according to the elements in the CP for CAP (issued by the NHC) related to antibiotic use [27]. Then, the auditors extracted information from the medical records corresponding to each item in the audit chart for each inpatient. To ensure the quality and consistency of the chart audit, we trained 5 auditors (master’s or PhD students with specialties in social medicine and health service management) on the meaning of each item on the checklist and how to judge adherence to antibiotic use according to the national CP for CAP. In addition, two experts were invited to review medical records from two hospitals that were reviewed by the auditors to analyse the consistency of the review between the experts and the auditors. The consistency rate between the auditors and the experts was 92.21%. One inspector was also assigned to check 10% of the reviewed charts in each hospital.

In addition, we asked the surveyed hospitals whether they had implemented the CP for CAP in their own hospitals and whether they provided CP training, initiated quality control for CP implementation and utilized incentive mechanisms for CP implementation.

### Data analysis

In the study, the compliance rate for the timely use of initial antibiotics reflected the proportion of inpatients who received initial antibiotics within 8 h after hospital admission, the compliance rate for the use of recommended antibiotics reflected the proportion of inpatients who received the recommended categories of antibiotics, the compliance rate for the use of the recommended antibiotic combination reflected the proportion of inpatients who received the recommended combination of antibiotics, and the overall compliance rate reflected the proportion of inpatients who received initial antibiotics from recommended categories and recommended combinations within 8 h [27]. Antibiotic use in emergency departments, outpatient departments or at home were not considered in the study because the national CP for CAP in China considers only inpatient care. For each indicator, the auditors judged whether a patient received recommended or non-recommended antibiotics and then assigned a code of “1” or “0”, respectively.

In the study, the patients with severe CAP were classified mainly by the hospital physicians in medical records or by the auditors according to the information in medical records and the criteria in the CAP guideline of China (2006) when the classification was not made by the hospital physicians. Severe CAP is defined in the CAP guideline of China (2006) as: a) having disturbance of consciousness; b) with ≥30 breaths per minute; c) with PaO_2_ < 60 mmHg, PaO_2_/FiO_2_ < 300 and/or mechanical ventilation needed; d) with arterial systolic pressure < 90 mmHg; e) with septic shock; f) bilateral or multiple pulmonary lobes involved and/or expanded lesion area ≥ 50% within 48 h of hospital admission shown in chest X-ray radiograph; or g) with oliguria (urine volume < 20 ml per hour or < 80 ml per 4 h), or acute renal failure requiring dialysis [6].

Chi-square tests and the t-test were used to compare differences in inpatient characteristics, antibiotic administration and compliance rates for antibiotic use between the hospitals that implemented the CP for CAP (CP group) and those that did not (non-CP group). A multivariate logistic analysis was used to test whether CP implementation in the hospitals influenced their overall compliance rates for antibiotic use while controlling for hospital (level and location) and inpatient characteristics (sex, age, number of comorbidities and CAP severity).

## Results

### Characteristics of the surveyed hospitals and CAP cases

In the 18 surveyed hospitals, the numbers of hospital beds in secondary and tertiary hospitals ranged from 249 to 910 and from 502 to 3283 in 2014, respectively. Among those hospitals, 12 hospitals implemented the CP for inpatients with CAP and conducted training and quality control for CP implementation. In addition, 9 hospitals that implemented the CP for inpatients with CAP also had incentives for CP implementation (Additional file 1: Table S1).

A total of 534 CAP cases were enrolled in the study, including 66.29% from the 12 hospitals that implemented the CP for CAP (CP group) and 33.71% from the 6 hospitals that did not implement the CP for CAP (non-CP group). Among the surveyed cases, the elderly (aged 60 years or older), males and patients with at least one comorbidity accounted for 46.63%, 48.13% and 61.42% of the sample, respectively. No significant differences were found in age, sex and the number of comorbidities between the patients in the CP and non-CP groups. However, the percentage of cases classified as severe CAP was higher in the non-CP group (19.38%) than in the CP group (7.26%) (*p* <  0.0001) (Table 1).

**Table 1:** Patient demographics in the CP and non-CP groups

Items	Total (*n* = 534)	CP group (*n* = 354)	Non-CP group (*n* = 180)	*p*-value^a^
Age (years)				
mean	56.31	55.47	57.96	0.147
SD	18.79	19.09	18.11	
Male				
n	257	174	83	0.506
%	48.13	49.15	46.11	
Comorbidities				
No comorbidity				
n	206	146	60	
%	38.58	41.24	33.33	
1 comorbidity				
n	141	87	54	0.176
%	26.40	24.58	30.00	
≥ 2 comorbidities				
n	187	121	66	
%	35.02	34.18	36.67	
Severe CAP ^b^				
n	53	22	31	< 0.0001
%	11.45	7.26	19.38	

^a^The t-test was used to compare the age of the inpatients with CAP and Chi-square tests were used to compare the other characteristics between the CP group and the non-CP group;

^b^Condition severities were not assessed for 71 inpatients

### Timely administration of initial antibiotics

The study showed that 84.18% of inpatients with CAP received initial antibiotics within 4 h of hospital admission, including 89.49% in the CP group and 73.74% in the non-CP group (*p* <  0.0001). According to the requirement of the CP for inpatients with CAP, 85.69% of the inpatients with CAP received initial antibiotics in a timely manner (within 8 h of hospital admission); this compliance rate was substantially higher in the CP group (90.63%) than in the non-CP group (75.98%) (*p* <  0.0001) (Table 2).

**Table 2:** Comparison of compliance rates for antibiotic use between the CP and non-CP groups

Items	Total (*n* = 531)		CP group (*n* = 352)		Non-CP group (*n* = 179)		*p*-value
	n	%	n	%	n	%	
Timely administration of antibiotics	455	85.69	319	90.63	136	75.98	< 0.0001
Use of recommended antibiotics	376	70.81	282	80.11	94	52.51	< 0.0001
One category of antibiotics used	239	83.28 ^a^	176	93.62	63	63.64	< 0.0001
Use of recommended combination of antibiotics	140	57.38	109	66.46	31	38.75	0.001
Overall compliance^b^	232	43.69	180	51.14	52	29.05	< 0.0001

^a^Compliance rates for the use of recommended antibiotics in inpatients with CAP who used only one antibiotic were significantly different from the compliance rates for the use of recommended combinations of antibiotics (*p* <  0.0001);

^b^Overall compliance applied to cases that met the requirements of timely use of initial antibiotics (≤ 8 h), use of recommended antibiotics and use of the recommended antibiotic combination

### Use of recommended antibiotics

**Fig. 1:**
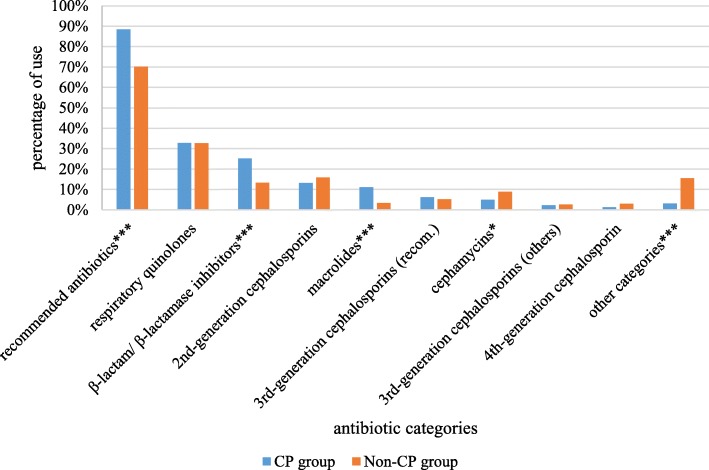
Distribution of antibiotic use in the CP and non-CP groups ^a^Recommended antibiotics include respiratory quinolones, β-lactam/β-lactamase inhibitors, 2nd-generation cephalosporins, macrolides and the recommended 3rd-generation cephalosporins (ceftriaxone and cefotaxime). *p < 0.05, ***p < 0.001.

A total of 18 categories of antibiotics (796 antibiotics) were administered to 531 inpatients with CAP from the 18 surveyed hospitals. Regarding the antibiotics administered to the inpatients with CAP, respiratory quinolones, β-lactam/β-lactamase inhibitors, 2nd-generation cephalosporins, macrolides and recommended 3rd-generation cephalosporins (ceftriaxone and cefotaxime) were among the top 5 most utilized antibiotics (accounting for 79.52% of the administered antibiotics) and were recommended in the CP for CAP in China. In addition, respiratory quinolones accounted for 32.79% of the antibiotics used in the inpatients with CAP, which was the most prescribed antibiotic in both the CP group (32.82%) and the non-CP group (32.72%), with no significant difference (*p* = 0.98) (Fig. 1). Approximately 79.52% of the 796 antibiotics administered to inpatients with CAP were the recommended antibiotics of the national CP.

The study also showed that 70.81% of the inpatients with CAP received the recommended antibiotics and that the compliance rate for the recommended antibiotics was much higher in the CP group (80.11%) than in the non-CP group (52.51%) (*p* <  0.0001) (Table 2).

### Use of the recommended combination of antibiotics

A total of 45.95% of the inpatients with CAP received combined antibiotics. The compliance rate for use of the recommended antibiotic combination was 57.38%, but it was much higher in the CP group (66.46%) than in the non-CP group (38.75%) (*p* = 0.001). In addition, the compliance rate for use of the recommended antibiotic combination (57.38%) was much lower than that for use of the recommended antibiotics for inpatients with CAP who received only one category of antibiotics (83.28%) (Table 2).

### Overall compliance with the CP

The overall compliance rate for inpatients with CAP was 43.69%; the overall compliance rate was much higher in the CP group (51.14%) than in the non-CP group (29.05%) (*p* <  0.0001) (Table 2).

### Factors influencing antibiotic use

A multivariate logistic model for the overall compliance among inpatients with CAP showed that the hospitals in the CP group had greater overall compliance than those in the non-CP group (odds ratio [OR] = 1.76; 95% confidence interval [CI] = 1.16–2.71) after controlling for hospital (level and location) and inpatient characteristics (sex, age, the number of comorbidities and severity). In addition, the hospitals in the Hubei and Gansu provinces had lower overall compliance (OR = 0.31, 95% CI = 0.20–0.48; OR = 0. 26, 95% CI = 0. 16–0.41) than those in Shanghai (Table 3).

**Table 3:** Multivariate logistic analysis of overall compliance with antibiotic use ^a^

Parameters	OR Estimates	95% CI ^b^		*p*-value
		Lower	Upper	
Intercept				0.760
Regions (comparison = Shanghai)				
Hubei province (1 = yes, 0 = no)	0.31	0.20	0.48	< 0.0001
Gansu province (1 = yes, 0 = no)	0.26	0.16	0.41	< 0.0001
Tertiary hospital (1 = yes, 0 = no)	0.73	0.50	1.07	0.108
Male (1 = yes, 0 = no)	0.93	0.64	1.35	0.699
Age (years)	1.01	1.00	1.02	0.184
No. of comorbidities (comparison = 0)				
1 comorbidity (1 = yes, 0 = no)	0.83	0.54	1.29	0.410
2 comorbidities (1 = yes, 0 = no)	1.05	0.60	1.83	0.871
Non-severe CAP (1 = yes, 0 = no)	0.85	0.54	1.32	0.463
Hospital implementation of CP (1 = yes, 0 = no)	1.76	1.16	2.71	0.009

^a^The dependent variable was overall compliance with antibiotic use in inpatients with CAP (1: overall compliance; 0: overall non-compliance), and χ^2^_likelihood ratio_ = 67.66, *p* < 0.001;

^b^CI: Confidence interval

## Discussion

This study found that CP implementation for patients with CAP has a positive influence on antibiotic utilization. However, some social and economic factors may affect CP compliance, which led to differences in CP compliance among hospitals in different regions of China.

### Relatively higher compliance with timely use of antibiotics

Timely initial use of antibiotics may lead to a lower risk of a fatal outcome in patients with CAP [28]. Our study found that antibiotic use in the inpatients with CAP was timely in most cases (84.18% received antibiotics within 4 h of hospital admission) and mostly followed the national CP (85.69% compliance rate). In the United States, the rates of administration of an initial dose of antibiotic within 4 and 8 h of admission were 78% and 93%, respectively [29]. However, the compliance rate for timely antibiotic use among inpatients with CAP was much higher in the CP group (90.63%) than in the non-CP group (75.98%). This discrepancy suggests that timely antibiotic use can be further improved if hospitals implement the CP for inpatients with CAP.

### Large gap in compliance with recommended antibiotics

To reach the goal of anti-infective therapy, appropriate antibiotics must be selected. In our study, we found that 796 antibiotics within 18 antibiotic categories were administered to inpatients with CAP. Respiratory quinolones, β-lactam/β-lactamase inhibitors, 2nd-generation cephalosporins, macrolides and 3rd-generation cephalosporins (ceftriaxone and cefotaxime), which were recommended in the CP for CAP in China, were among the top 5 utilized antibiotics (approximately 80% of the antibiotics used in inpatients with CAP).

In particular, respiratory quinolones accounted for approximately one-third of the antibiotics used in both the CP and non-CP groups in the study, which may be attributed to their effective antibacterial activity, less prominent cross-drug resistance, low protein-binding rate and high capacity for tissue penetration [30]. In China, a number of epidemiological studies have found that mycoplasma pneumoniae and streptococcus pneumoniae are important pathogens of adult CAP [31–35]. Furthermore, mycoplasma pneumoniae is highly resistant to macrolides but is sensitive to doxycycline or minocycline and quinolones [36, 37], which may be one of the reasons that in China, respiratory quinolones are the recommended medications in the CAP guidelines [6, 38]. In fact, respiratory quinolones are also generally recommended for non-ICU hospitalized CAP patients in the guidelines of many other countries [3, 5, 39]. However, respiratory quinolones are broad-spectrum antibiotics, and the use of them in initial treatments for CAP patients may lead to a delay in the discovery of and implementation of appropriate therapy for pulmonary tuberculosis [40–43]; therefore, caution should be employed when respiratory quinolones are used as an initial therapy for CAP.

In addition, the study revealed that 70.81% of the inpatients with CAP were administered the recommended antibiotics, which was lower than the rate in the United States (81%) [29]. A large gap existed in compliance with the use of recommended antibiotics between the CP (80.11%) and non-CP groups (52.51%).

### Low compliance with the recommended combination of antibiotics

Appropriate antibiotic use can improve antibacterial effectiveness and can reduce side effects in inpatients with CAP [44]; therefore, appropriate antibiotic use also includes the appropriate use of combined antibiotics. Our study revealed that 45.95% of the inpatients with CAP received combined antibiotics in hospitals in China and that the compliance rate for the use of the recommended combination of antibiotics was much lower than that in the inpatients who received non-combined antibiotics (57.38% vs. 83.28%). This finding suggested that many inpatients with CAP did not receive appropriate combined antibiotics according to the recommendations in the national CP. Inappropriate use of combined antibiotics in China was also found in one study of primary healthcare facilities in China [11].

### Facilitation of CP implementation for better antibiotic utilization

Our study demonstrated a large gap between evidence-based recommended therapy and actual clinical practice [45, 46]. The overall compliance rate in China (43.69%) was much lower than the 75–80% compliance rates reported for antibiotic prescriptions for CAP patients in some previous studies [29, 47, 48]. Inappropriate antibiotic utilization can not only impact the effectiveness and safety of medical care for CAP patients but can also easily create multidrug-resistant bacterial strains [48].

Adherence to CAP guidelines has been shown to improve clinical outcomes, including reductions in the length of hospital stay, morbidity and mortality [49, 50]. The CP for CAP in China is based on CAP guidelines; therefore, antibiotic use consistent with the CP can theoretically result in more effective and safer patient care. This study showed that inpatients with CAP in the CP group received antibiotics more concordantly with the CP (51.14% vs. 29.05% overall compliance). The multivariate logistic analysis also found that the hospitals that implemented the CP had greater overall compliance than the hospitals that did not (OR = 1.76) after controlling for hospital and inpatient characteristics. Our study indicated that facilitation of CP implementation improved antibiotic utilization, including initial antibiotic administration, antibiotic selection and the use of combined antibiotics.

Although the 12 hospitals that implemented the CP for inpatients with CAP in our study had training and quality control for CP implementation, 3 hospitals did not have incentives for CP implementation. Many articles have discussed the effects of social, organizational, cognitive and/or motivational factors on the implementation of clinical practice guidelines (CPGs) or CPs [51–53]. Other factors, such as CP quality, which is mainly related to whether the CP is applied in complex clinical practice in a timely, effective and accurate manner, and internal and external support from the hospital, may also affect CP implementation.

### Associations of local social and economic statuses with antibiotic use

According to the findings from the multivariate logistic analysis, the hospitals in Shanghai, which is one of the most developed cities in China, had a higher overall compliance rate for antibiotic use than those in the Hubei and Gansu provinces, suggesting that external social and economic statuses affected antibiotic use. One probable reason for this result was greater awareness of the negative impacts of inappropriate antibiotic use on patient outcomes among hospitals, physicians and the local population and strict monitoring of antibiotic use by the local government in Shanghai [54, 55]. Another possible reason is that hospital regions with higher social and economic levels tend to allocate more resources towards improving antibiotic utilization (including more education on the proper use of antibiotics and the construction of health information systems to provide antibiotic information, authorize antibiotic prescriptions, warn against unusual utilization and provide real-time monitoring) and providing financial incentives for proper antibiotic use based on related assessments [56–58].

## Conclusions

In China, the overall compliance rate with the national CP for inpatients with CAP was low, but the inpatients in the hospitals of the CP group received antibiotics more concordantly with the national CP. Adherence to evidence-based care has been shown to improve clinical outcomes but requires internal and external support from hospitals to facilitate CP implementation for inpatients with CAP and governmental commitment, hospital input and population involvement to improve antibiotic utilization.

## Additional file

Additional file 1: **Table S1.** CP implementation in the surveyed hospitals. (DOCX 17 kb)

## Abbreviations

CAP: Community-acquired pneumonia
CGs: Clinical guidelines
CI: Confidence interval
CP: Clinical pathway
CPG: Clinical practice guidelines
ICD-10: International Statistical Classification of Diseases and Related Health Problems 10th Revision
ICU: Intensive care unit
IDSA: Infectious Diseases Society of America
NHC: National Health Commission
OR: Odds ratio
WHO: World Health Organization
