# Supplementary material for: Effects of a clinical pathway on antibiotic use in patients with community-acquired pneumonia: a multi-site study in China
Source: BMC Infect Dis. 2018 Sep 19;18:471. doi: 10.1186/s12879-018-3369-1 (PMC6146630; doi:10.1186/s12879-018-3369-1)
Supplement: Supplementary file 1 — Table S1. CP implementation in the surveyed hospitals. (DOCX 17 kb) [file 12879_2018_3369_MOESM1_ESM.docx]

Table S1 CP implementation in the surveyed hospitals ^a^

| Hospital | CP training | Quality control for CP implementation | Incentives for CP implementation | CP implementation for CAP inpatients |
| --- | --- | --- | --- | --- |
| 1 | 1 | 1 | 1 | 1 |
| 2 | 1 | 1 | 1 | 1 |
| 3 | 1 | 1 | 1 | 0 |
| 4 | 1 | 1 | 0 | 1 |
| 5 | 1 | 1 | 1 | 1 |
| 6 | 1 | 1 | 1 | 1 |
| 7 | 1 | 1 | 1 | 0 |
| 8 | 1 | 1 | 1 | 1 |
| 9 | 1 | 1 | 1 | 1 |
| 10 | 1 | 1 | 1 | 1 |
| 11 | 1 | 1 | 1 | 1 |
| 12 | 0 | 0 | 0 | 0 |
| 13 | 1 | 1 | 1 | 1 |
| 14 | 1 | 1 | 0 | 1 |
| 15 | 0 | 0 | 0 | 0 |
| 16 | 1 | 1 | 1 | 0 |
| 17 | 1 | 1 | 1 | 0 |
| 18 | 1 | 1 | 0 | 1 |

^a^ 1 indicates the presence of CP training, quality control for CP implementation, incentives for CP implementation or CP implementation for CAP inpatients, whereas 0 indicates the absence of these variables.
